# Immunoregulatory role of hesperidin against ovalbumin (OVA)-induced bronchial asthma and depression in rats

**DOI:** 10.1007/s00210-023-02833-7

**Published:** 2023-11-11

**Authors:** Abeer Salama, Mona S. O. Gouida, Noha N. Yassen, Ahmed A. Sedik

**Affiliations:** 1 Pharmacology Department, Medical Research and Clinical Studies Institute, National Research Centre, El-Buhouth St., Dokki, Cairo, 12622 Egypt; 2 Genetics Unit, Faculty of Medicine, Children Hospital, Mansoura University, Mansoura, Egypt; 3 Pathology Department, National Research Centre, El-Buhouth St., Dokki, Cairo, 12622 Egypt

**Keywords:** Hesperidin, Ovalbumin, Bronchial asthma, Depression

## Abstract

Links between bronchial asthma and depression have recently become a great subject of interest. The present study was carried out to assess the protective role of hesperidin against ovalbumin (OVA)-induced bronchial asthma that is associated with depression in rats, for this purpose, four groups. Rats were sensitized with intraperitoneal administration of 200 μg OVA/10 mg aluminum hydroxide (Al (OH) 3 for 3 consecutive days then at day 11 followed by intranasal challenge with OVA (1.5 mg/kg) at days 19, 20, and 21. Rats were pretreated with hesperidin (100 & 200 mg/kg) 1h before OVA challenge. At the end of the study, behavioral tests, biochemical indices, and histopathological architectures of lung and brain tissues were evaluated. Our findings showed that hesperidin significantly ameliorated the reduction in motor activity, motor coordination, forced swimming, CD4, CD25 and foxp3, interleukin-10 (IL-10), dopamine, serotonin, and neurotrophin-3 (NT3) as well as alleviated the elevation in transforming growth factor-beta (TGF-β), tumor necrosis factor-alpha (TNF-α), iL-5, and immunoglobulin E (IgE). In addition, hesperidin reduced cellular infiltration, alveolar sacs damage, the bronchioles wall disruption, and nuclei pyknosis in neuron cells. Finally, hesperidin may provide protection against OVA-induced asthma and depression. This impact could be mediated in part by its anti-inflammatory and immunoregulatory properties.

**Graphical abstract:**

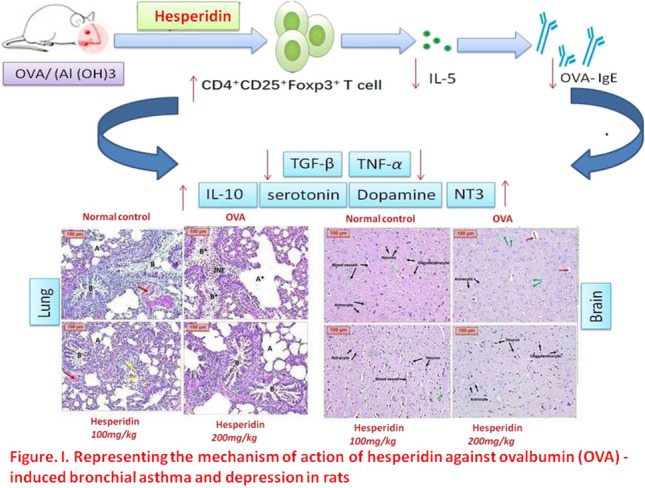

## Introduction

Bronchial asthma is a chronic airway inflammatory condition that causes unexpected episodes of shortness of breath and air flow obstruction (Kelly, 2022). The prevalence rate of asthma has risen recently in a dramatic manner and affects approximately 235 million people worldwide due to the massive exposure to chemicals, pollen, dust, and industrial pollutants (Ko et al., 2020). Asthma is characterized by eosinophilic airway inflammatory response, secretion of allergen-specific immunoglobulin E (IgE) with excessive secretion of mucous that leads to remodeling and hyperresponsiveness in the airways (Andreone, 2023). Asthmatics are more likely to experience stress and negative emotions such as fear, panic, irritability, bipolar disorder, and depression symptoms compared with a normal population (Zielinski et al., 2000, Opolski and Wilson, 2005, Smith et al., 2006).

Prior studies have postulated that asthma could be attributed to a shift in the T helper Th1/Th2 balance and a shift in the ratio of regulatory T cells (Chen et al., 2013). An increase in the production of IgE and Th2 cytokines with accumulation of eosinophils in the lungs is the most distinguishable features in asthmatic patients (Kim et al., 2011). It has been documented that interleukin-5 (IL-5) plays a central role in the regulation of eosinophilic inflammation in asthmatic patients (Renauld, 2001). Recent studies have shown the role of transforming growth factor (TGF)-β and interleukin-10 (IL-10) in controlling Th2 responses for the treatment of bronchial asthma (Barnes, 2001, Nakao, 2001). Although corticosteroids are the most potent non-specific anti-inflammatory therapies and are frequently used to treat asthma, it has been observed that they can induce systemic immunosuppression, which enhances the risk of further infections and relapse is a common problem after therapy withdrawal (Sheth et al., 2006, Lloyd and Hawrylowicz, 2009). Furthermore, corticosteroids do not alter the progression of asthma due to their mild effects on the structural changes in the airways (Jeffery et al., 2012). As a result, our study was directed to find safe and effective alternatives especially from natural sources, hence hesperidin (4′-methoxy-7-O-rutinosyl-3′,5- dihydroxyflavanone), a specific flavonoid glycoside that is frequently present in citrus species, such as grapefruit, lemon, and orange. It has been reported to exert a wide range of pharmacological effects that include antioxidant, anti-inflammatory, anticancer, and antidepressant-like actions (Hajialyani et al., 2019, de Souza et al., 2022). In addition, it is a treasure trove due to its safety profile, non-accumulative nature, and restricted side effects, even during pregnancy (Garg et al., 2001, Devi et al., 2015). Intriguingly, after referring to the previous studies, it was found that there was no relevant study about the protective role of hesperidin against OVA-induced asthma and related disorders such as depression in rat’s model.

## Materials and methods

### Animals

Twenty-four adult male Wister albino rats weighing 130–150 g were obtained from the colony of National Research Centre (NRC), Egypt. Rats were housed in controlled environments with unrestricted access to a typical commercial pellet diet and water, as well as controlled environmental conditions for temperature (22±2°C), humidity (60%), and normal photoperiod (12–12 h light-dark cycles). The present study was carried out in accordance with the pertinent guidelines and regulations, which were authorized by the Medical Research Ethics Committee, NRC, Egypt (approval number; 92311122022). In addition, the research protocol adhered to the National Institutes of Health’s guidelines for the use and care of laboratory animals. The present animal experiment was adhered to the ARRIVE guidelines.

### Chemicals

Ovalbumin (OVA; CAS number: 138831/86/4), hesperedin (CAS number: 520-26-3), and aluminum hydroxide (Al (OH) 3; CAS number: 21645/51/2) were obtained from Sigma Aldrich Chemical Co. (USA).

### Induction of bronchial asthma

Bronchial asthma was induced by intraperitoneal (i.p.) administration of 200 μg OVA/10 mg (Al (OH)_3_ suspended in 1 mL normal saline on days 1, 2, 3, and 11) then intranasal administration of OVA (1.5 mg/kg) at days 19, 20, and 21 of the study (Abdel-Fattah et al., 2015, Sherkawy et al., 2018).

### Experimental design

**Fig. 1:**
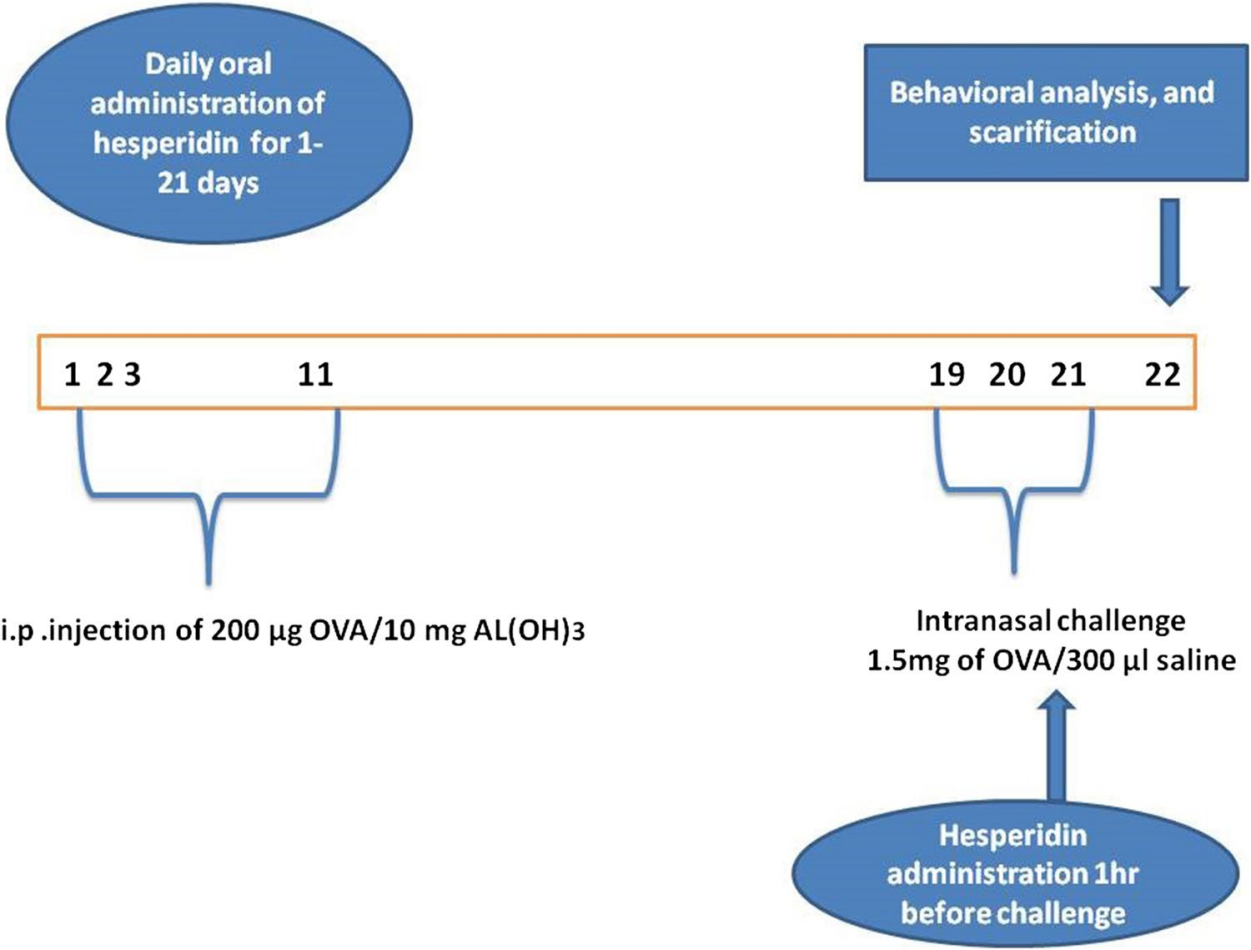
Schematic representation of the experimental study

After adaptation for 2 weeks, rats were allocated into four groups (*n*= 6). In group 1, rats were given only normal saline i.p, intranasal and p.o. Bronchial asthma was induced in the other three groups that were distributed as follows: Group 2 was kept untreated and regarded as asthmatic control group. Groups 3 and 4 were given hesperidin 100 and 200 mg/kg orally 1 h before OVA challenge at days 19, 20, and 21 (Fig. 1). The doses of hesperidin were selected according to previous study (Hasanin et al., 2020).

#### Behavioral indices

Behavioral parameters were evaluated 18h after OVA challenge and oral dosing with hesperidin.

##### Assessment of motor activity

Infrared photocell principle was utilized to count the motor activity of rats by using an activity cage (Ugo-Basile, Model 7430, Italy) (Marazioti et al., 2005). Rats were acclimatized to the test room for 1 h before placing it in the activity cage (exposure). The activity counts were pretested in three successive sessions; each was of 5 min duration before starting experiment to habituate them to the apparatus. Then, the rats were placed in the activity cage and the activity counts of each rat were calculated over 5 min duration (Sedik et al., 2023).

##### Assessment of motor coordination

An accelerating rotarod (UgoBasile, Model 7750, Italy) was adopted to assess the motor performance of rats that were subjected to previous training (Afifi et al., 2021, Sedik and Elgohary, 2023). Rats were first habituated to the stationary rod for 5 min. Rats were subjected to a training of three sessions on three consecutive days on the rotarod apparatus using accelerating speed that was initiated at 4 rpm and increased gradually to reach 40 rpm over 300 s. At the end of experiment, rats were then placed on the rod, starting at 4 rpm and accelerated linearly to 40 rpm over 300 s. Rotarod performance of rats was calculated as the average time in seconds spent on the rod (Salama and Elgohary, 2021).

##### Forced swimming test 

Forced swimming test (FST) was conducted in a glass cylinder filled with enough water (temperature; 23±1°C) to prevent the rats’ hind limbs from touching the bottom (40 cm high, 18 cm diameter). One hour after last treatment with hesperidin 100 and 200 mg/kg, the time of swimming was estimated (Cryan et al., 2005).

### Euthanasia and preparation of lung and brain homogenate

Rats were sacrificed by cervical dislocation after the last behavioral test, and their lungs and brains were gently dissected then washed with saline where 20% homogenate of the lung and brain from different groups were centrifuged at 3000 ×g (4°C) for 15 min. The stored lung and brain supernatants in −80°C were evaluated by flow cytometry and ELISA techniques.

#### Evaluation of TNF-α, IL-5, and IgE in lung homogenate

Levels of TNF-α and IL-5 were determined in lung homogenate using SunLong Biotec Co., LTD, China (catalogue. no. SL0722Ra), SunRed Biotechnology, Company, China (catalogue. no. 201-11-0135) ELISA kits, respectively, as described by the manufacturer, and their levels were expressed as ng/L and pg/mL, respectively. In addition, the levels of total IgE were determined in lung homogenate by SunRed Biotechnology, Company, China ELISA kit (catalogue. no. 201-11-0453), as described by the manufacturer and its level was expressed as (KU/L).

#### Evaluation of dopamine, serotonin, and NT3 in brain homogenate

Levels of dopamine, serotonin, and NT3 were determined in brain homogenate using Sunlong Biotech Co., LTD, Shanghai, China ELISA kits (catalogue. no SL0243Ra, SL1046Ra and SL0522Ra), respectively, as described by the manufacturer.

#### Evaluation of TGF-β, IL-10, CD3, and treg (CD4, CD25, and Foxp3) in lung and brain homogenate

Lung and brain tissues were dissected gently under complete aseptic conditions, where they were incubated under optimum temperature after being diluted in phosphate buffer saline (PBS), then cells were dispersed, collected, centrifuged at 400 ×*g* for 4–5 min at 2–8°C and resuspended again in PBS. Finally, the cell pellets re-centrifuged again and resuspended in an appropriate volume of flow cytometry staining buffer to be ready for conjugation with specific antibodies (Holmes et al., 2001).

The prepared cells from lung specimens were conjugated with rat TGF-β-RII PE-antibody (catalogue number FAB532P) and FITC rat monoclonal il-10 antibody (catalogue number 556923). The prepared cells from brain specimens were conjugated with rat anti-CD4 (catalogue number 554837), anti-CD25 (catalogue number 550616), and Foxp3 (catalogue number 71577549). Cell damage was assessed by using a BD-Acuuri-C6 flow cytometer (BD Biosciences, San Jose, CA, USA), and the data were analyzed using Cell Quest v3.3 software (Bajgelman, 2019).

#### Evaluation of the histopathological architecture of the lung and brain specimens

Lung and brain specimens were dissected instantly, and fixed in 10% neutral-buffered formalin solution, where the specimens were washed, dehydrated in alcohol, and cleared in xylene and embedded in paraffin**.** The embedded paraffin specimens were cut (3 μm thick) and stained with hematoxylin and eosin to be visualized by using a light microscope (Carleton et al., 1980).

### Statistical analysis

All quantifiable comparisons in our research were made using one-way analysis of variance (ANOVA), and Tukey’s multiple comparison test was carried out using the GraphPad Prism program 8.0, USA. Results are shown as the mean ± SD of (6 rats), and the difference was recognized significant when *p* value is ≤ 0.05.

## Results

### Effect of hesperidin on the behavioral indices in rats received OVA-induced bronchial asthma and depression

**Fig. 2:**
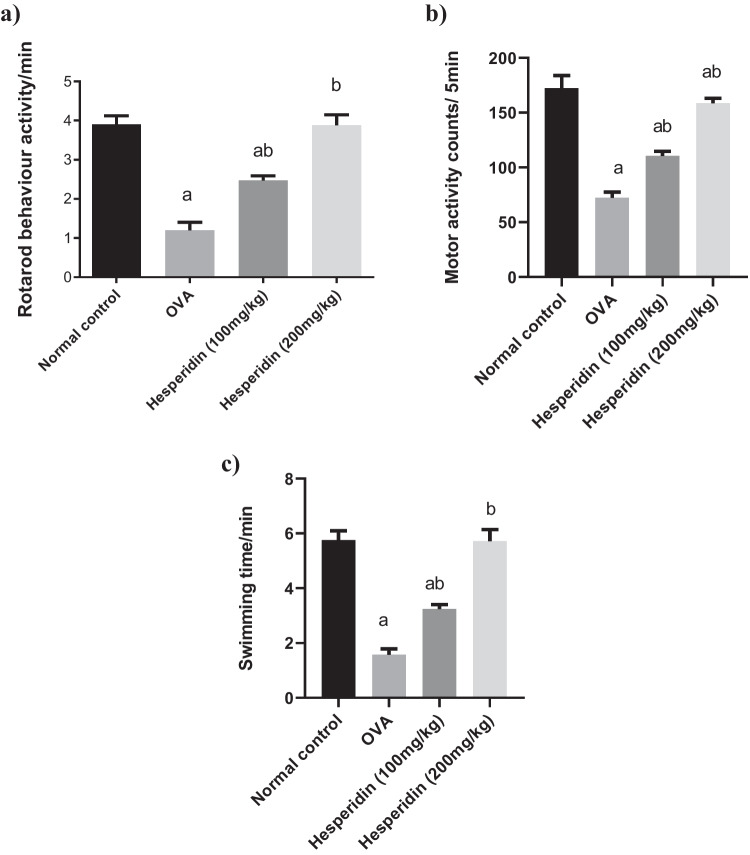
Effect of hesperidin on (**a**) rotarod behavior activity, (**b**) motor activity, and (**c**) swimming time in OVA-induced bronchial asthma and depression in rats. Asthma was induced by i.p. administration of 200 μg OVA/10 mg aluminum hydroxide suspended in 1 mL normal saline for 3 consecutive days then intranasal OVA at days 19, 20, and 21. Hesperidin was orally administered from day 1 and 1 h before each OVA challenge. 18 h after OVA challenge and oral dosing with hesperidin. Rotarod,activity and forced swimming tests were evaluated. a Significantly different from normal control at *p*<0.05. b Significantly different from OVA group at *p*<0.05

Rats received OVA and were manifested by a significant decrease in rotarod activity, motor count, and FST, by 0.3-folds, 0.41-folds, and 0.27-folds, respectively, as compared to normal values. Rats were pretreated with hesperidin (100 mg/kg) and revealed a marked increase in those parameters, by twofolds, 1.5-folds, and twofolds, respectively, as compared to OVA-received group, while rats that were pretreated with hesperidin (200 mg/kg) revealed a marked increase in the previous parameters, by threefolds, twofolds, and 3.5-folds, respectively, as compared to OVA-received group (Fig. 2).

### Effect of hesperidin on the lung levels of TNF-α, IL-5, and IgE in rats that received OVA-induced bronchial asthma and depression in rats

**Fig. 3:**
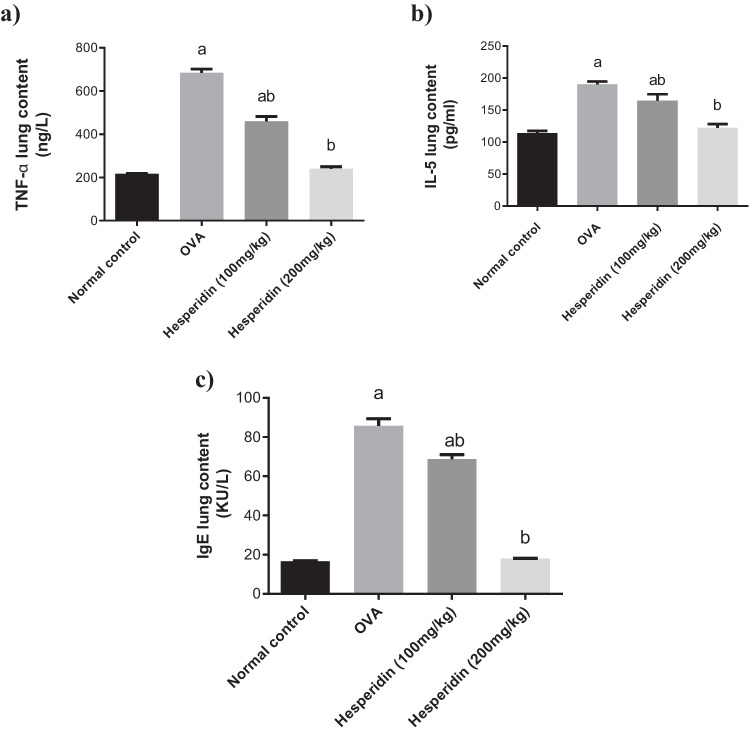
Effect of hesperidin on (**a**) lung tumor necrosis factor-alpha (TNF-α), (**b**) lung interleukin (il-5), and (**c**) lung immunoglobulin-E (Ig-E) in OVA-induced bronchial asthma and depression in rats. Asthma was induced by i.p. administration of 200 μg OVA/10 mg aluminum hydroxide suspended in 1 mL normal saline for 3 consecutive days then intranasal OVA at days 19, 20, and 21. Hesperidin was orally administered from day 1 and 1 h before each OVA challenge. Lung tissue samples were collected 24h after the last challenge to evaluate the levels of TNF-α, il-5, and Ig-E. a Significantly different from normal control at *p*<0.05. b Significantly different from OVA group at *p*<0.05

Lung levels of TNF-α, IL-5, and IgE in rats that received OVA were significantly increased by threefolds, 1.6-folds, and fivefolds, respectively, as compared to normal group. Rats pretreated with hesperidin (100 mg/kg) revealed a marked decrease in those parameters, by 0.67-folds, 0.86-folds, and 0.80-folds, respectively, as compared to OVA-received group, while rats that were pretreated with hesperidin (200 mg/kg) revealed a marked decrease in the previous parameters, by 0.35-folds, 0.64-folds, and 0.21-folds, respectively, as compared to OVA received group (Fig. 3).

### Effect of hesperidin on TGF-β and IL-10 lung content in OVA-induced bronchial asthma and depression in rats

**Fig. 4:**
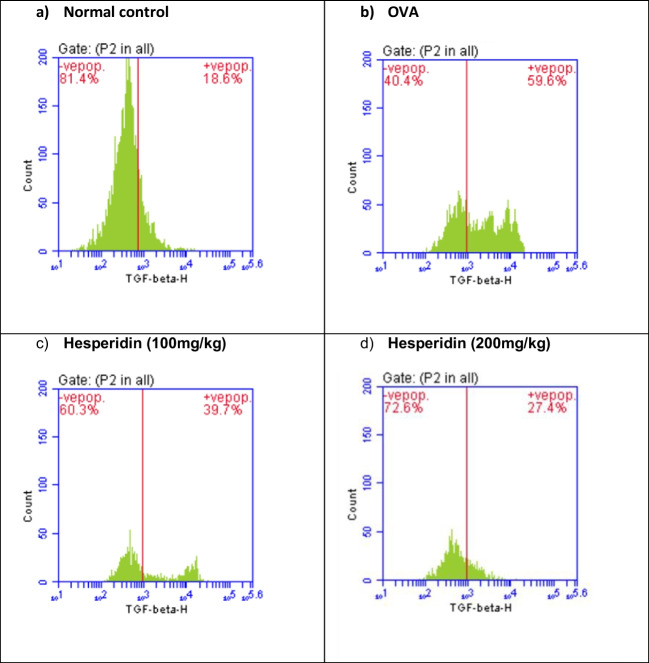
Effect of hesperidin on TGF-β lung content in OVA-induced bronchial asthma and depression in rats. Asthma was induced by i.p. administration of 200 μg OVA/10 mg aluminum hydroxide suspended in 1 mL normal saline for 3 consecutive days then intranasal OVA at days 19, 20, and 21. Hesperidin was orally administered from day 1 and 1 h before each OVA challenge. Lung tissue samples were collected 24h after the last challenge to evaluate TGF-beta levels. Flow cytometry histogram of OVA, showing higher level of TGF-β (59.5%) than in normal control (18.6%), while hesperidin (100mg/kg) and hesperidin (200mg/kg) showing lower level of TGF-β (39.7% and 27.4%), respectively, than in OVA

**Fig. 5:**
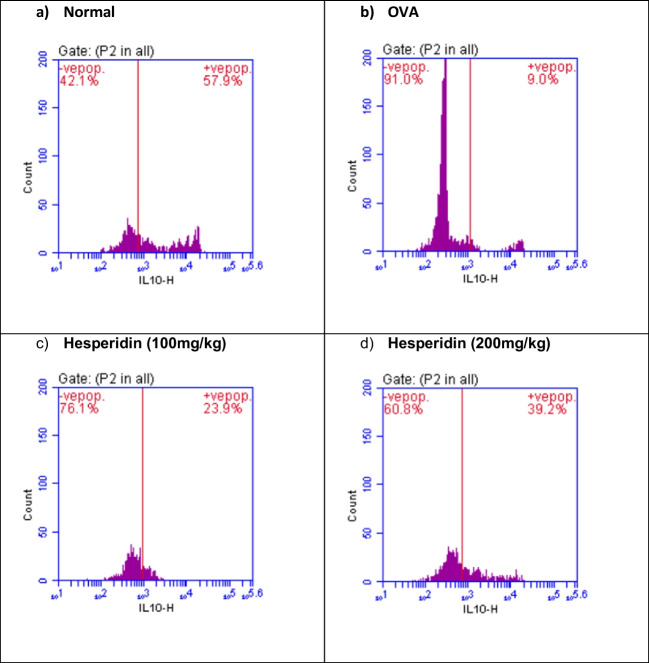
Effect of hesperidin on il-10 lung content in OVA-induced bronchial asthma and depression in rats. Asthma was induced by i.p. administration of 200 μg OVA/10 mg aluminum hydroxide suspended in 1 mL normal saline for 3 consecutive days then intranasal OVA at days 19, 20, and 21. Hesperidin was orally administered from day 1 and 1 h before each OVA challenge. Lung tissue samples were collected 24h after the last challenge to evaluate il-10 levels. Flow cytometry histogram of OVA, showing lower level of il-10 (9%) than in normal control (57.9%), while hesperidin (100mg/kg) and hesperidin (200mg/kg) showing higher level of il-10 (23.9% and 39.2%), respectively, than in OVA

The rats that received OVA were associated with a significant increase by threefolds in the lung levels of TGF-β and a significant reduction in IL-10 by 0.15-folds, as compared to normal group. Rats pretreated with hesperidin (100 mg/kg) revealed a marked decrease in TGF-β by 0.66-folds and an increase in IL-10 by 2.6-folds, as compared to OVA-received group, while rats that were pretreated with hesperidin (200 mg/kg) revealed a normalized values in the previous parameters (Figs. 4 and 5).

### Effect of hesperidin on the brain levels of serotonin, dopamine, and neurotrophin-3 in OVA-induced bronchial asthma and depression in rats

**Fig. 6:**
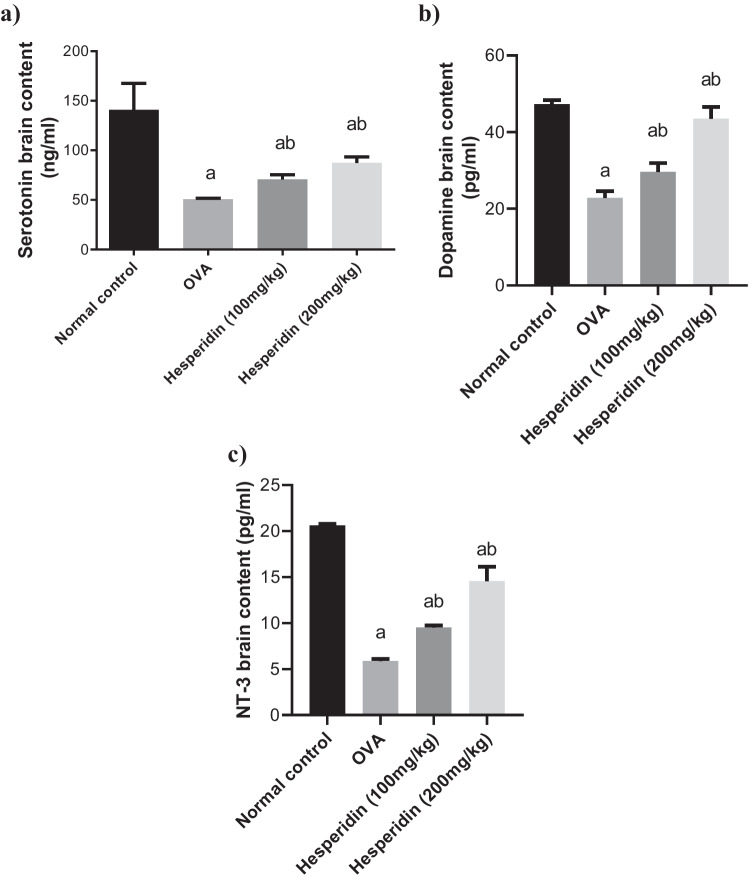
Effect of hesperidin on (**a**) brain serotonin, (**b**) brain dopamine, and (**c**) brain NT3 in OVA-induced bronchial asthma and depression in rats. Asthma was induced by i.p. administration of 200 μg OVA/10 mg aluminum hydroxide suspended in 1 mL normal saline for 3 consecutive days then intranasal OVA at day 19, 20, and 21. Hesperidin was orally administered from day 1 and 1 h before each OVA challenge. Brain tissue samples were collected 24h after the last challenge to evaluate the levels of serotonin, dopamine, and NT3. a Significantly different from normal control at *p*<0.05. b Significantly different from OVA group at *p*<0.05

Brain levels of serotonin, dopamine, and NT-3 in rats that received OVA were significantly decreased by 0.35-folds, 0.48-folds, and 0.28-folds, respectively, as compared to normal group. Rats pretreated with hesperidin (100 mg/kg) revealed a marked increase in those parameters, by 1.39-folds, 1.29-folds, and 1.62-folds, respectively, as compared to OVA-received group, while rats that were pretreated with hesperidin (200 mg/kg) revealed a marked increase in the previous parameters, by 1.72-folds, 1.90-folds, and 2.4-folds, respectively, as compared to OVA-received group (Fig. 6).

### Effect of hesperidin on CD4, CD25, and Foxp3 brain content in OVA-induced bronchial asthma and depression in rats

**Fig. 7:**
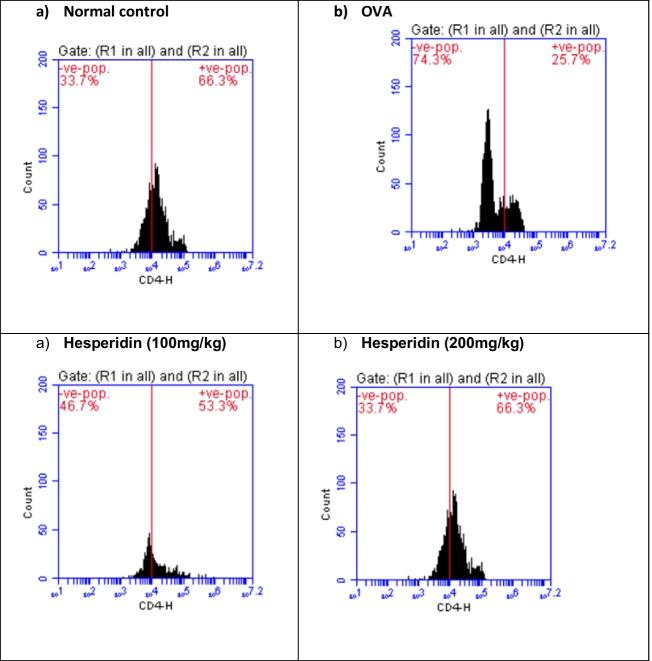
Effect of hesperidin on CD4 brain content in OVA-induced bronchial asthma and depression in rats. Asthma was induced by i.p. administration of 200 μg OVA/10 mg aluminum hydroxide suspended in 1 mL normal saline for 3 consecutive days then intranasal OVA at days 19, 20, and 21. Hesperidin was orally administered from day 1 and 1 h before each OVA challenge. Brain tissue samples were collected 24h after the last challenge to evaluate CD4 levels. Flow cytometry histogram of OVA, showing lower level of CD4 (25.7%) than in normal control (66.3%), while hesperidin (100mg/kg) and hesperidin (200mg/kg) showing higher level of CD4 (53.3% and 66.3%), respectively, than in OVA

**Fig. 8:**
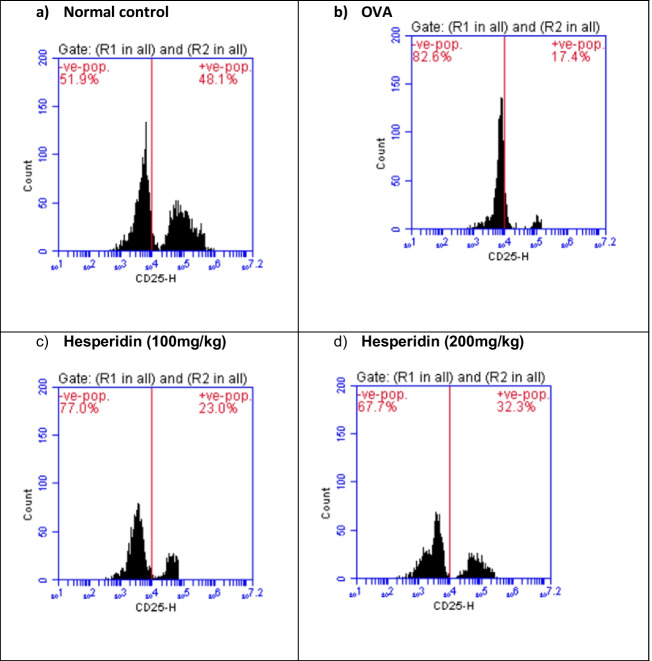
Effect of hesperidin on CD25 brain content in OVA-induced bronchial asthma and depression in rats. Asthma was induced by i.p. administration of 200 μg OVA/10 mg aluminum hydroxide suspended in 1 mL normal saline for 3 consecutive days then intranasal OVA at days 19, 20, and 21. Hesperidin was orally administered from day 1 and 1 h before each OVA challenge. Brain tissue samples were collected 24h after the last challenge to evaluate CD25 levels. Flow cytometry histogram of OVA, showing lower level of CD25 (17.4%) than in normal control (48.1%), while hesperidin (100mg/kg) and hesperidin (200mg/kg) showing higher level of CD25 (23% and 32.3%), respectively, than in OVA

**Fig. 9:**
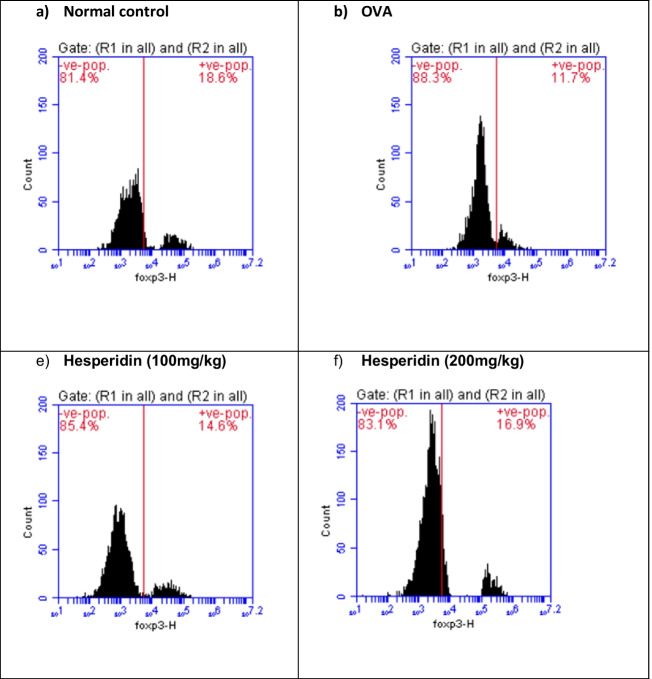
Effect of hesperidin on Foxp3 brain content in OVA-induced bronchial asthma and depression in rats. Asthma was induced by i.p. administration of 200 μg OVA/10 mg aluminum hydroxide suspended in 1 mL normal saline for 3 consecutive days then intranasal OVA at days 19, 20, and 21. Hesperidin was orally administered from day 1 and 1 h before each OVA challenge. Brain tissue samples were collected 24h after the last challenge to evaluate Foxp3 levels. Flow cytometry histogram of OVA, showing lower level of Foxp3 (11.7%) than in normal control (18.6%), while hesperidin (100mg/kg) and hesperidin (200mg/kg) showing higher level of Foxp3 (14.6% and 16.9%), respectively, than in OVA

Brain levels of CD4, CD25, and Foxp3 in rats that received OVA were significantly decreased by 0.38-folds, 0.36-folds, and 0.63-folds, respectively, as compared to normal group. Rats pretreated with hesperidin (100 mg/kg) revealed a marked increase in those parameters, by twofolds, 1.3-folds, and 1.2-folds, respectively, as compared to OVA-received group, while rats that were pretreated with hesperidin (200 mg/kg) revealed a significant increase in the previous parameters by 2.6-folds, 1.85-folds, and 1.44-folds, respectively, as compared to OVA-received group (Figs. 7, 8, and 9).

### Effect of hesperidin on the histopathological alterations in the lung tissues of OVA-induced bronchial asthma and depression in rats

**Fig. 10:**
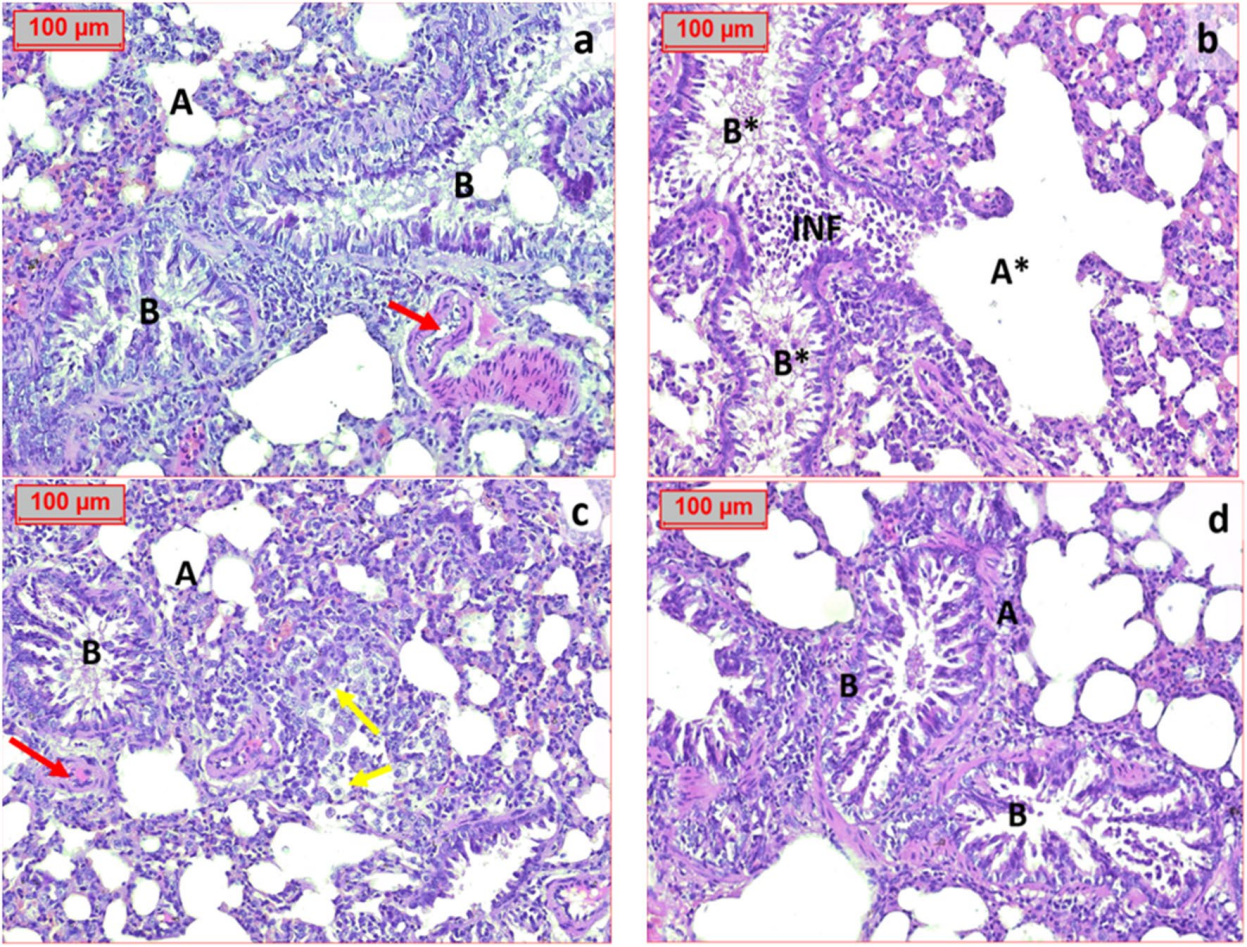
Effect of hesperidin on the histopathological changes in the lung tissues of OVA-induced bronchial asthma and depression in rats. A photomicrography of lung tissue in normal control group (**a**) showing normal looking architecture, the alveolar sacs (A), separated by alveolar septa, comprising alveolar lining cells and thin walled capillaries. The bronchiole (B) have intact wall with normal looking ciliated columnar epithelium, normal looking bronchial vessels (eed arrows). OVA group (**b**) showing the alveolar sacs totally destructed (A*). The bronchiole (B*) have damaged wall by inflammatory cells infiltrate (INF). Hesperidin (100mg/kg) (**c**) showing the alveolar sacs (A), separated by alveolar septa, infiltrated by inflammatory cells. The bronchiole (B) have intact wall with normal looking ciliated columnar epithelium; other areas have inflammatory cells infiltrate with prominent macrophages (yellow arrows), normal looking bronchial vessels (red arrows). Hesperidin (200mg/kg) (**d**) showing normal looking architecture, the alveolar sacs (A), separated by alveolar septa, comprising alveolar lining cells and thin walled capillaries. The bronchiole (B) have intact wall with normal looking ciliated columnar epithelium (H&E ×200)

A photomicrography of lung tissue in normal control group revealed normal histopathological picture. Lung tissue sections from OVA group showed severe cellular infiltration, damaged alveolar sacs, and disruption in the wall of bronchioles. Lung tissue section of a rat pretreated with hesperidin (100 mg/kg) showed less damaged alveolar sacs with few inflammatory cells, while rats that were pretreated with hesperidin (200 mg/kg) revealed a significant improvement in the looking architecture of lung tissues (Fig. 10).

### Effect of hesperidin on the histopathological alterations in the brain tissues of OVA-induced bronchial asthma and depression in rats

**Fig. 11:**
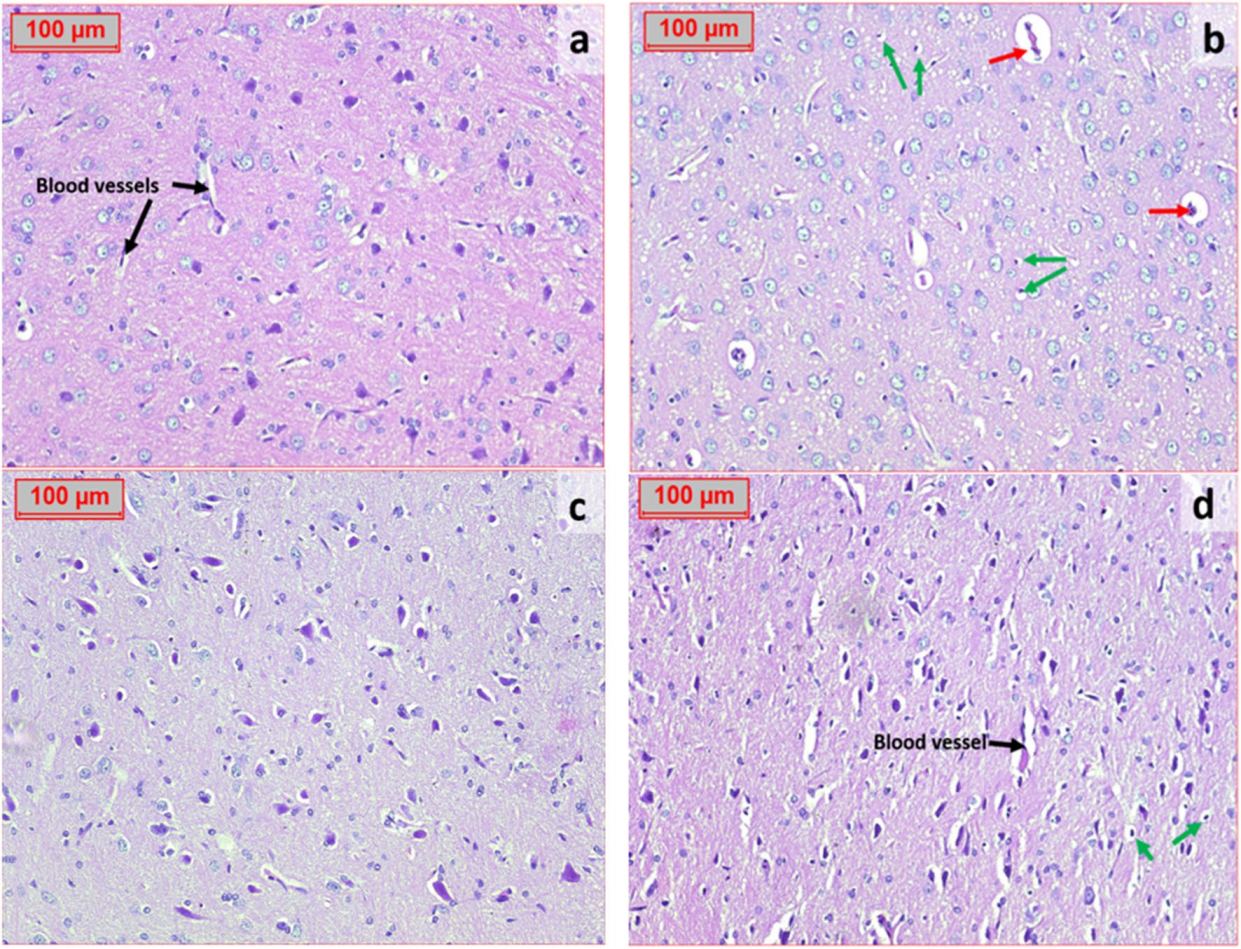
Effect of hesperidin on the histopathological changes in the brain tissues of OVA-induced bronchial asthma and depression in rats. A photomicrography of brain cortex in normal control group (**a**) showing normal structure of cerebral cortex formed of neuron cells, typically have large pale nuclei with prominent nucleoli. The non-neuronal cells (glial) cells include oligodendrocytes; hyperchromatic round nuclei with abundant clear cytoplasm, astrocytes; paler more elongated nuclei with scant cytoplasm; and normal looking blood vessels. OVA group (**b**) showing most of neuron cells have pyknotic nuclei (green arrows), while astrocytes have normal looking paler more elongated nuclei with scant cytoplasm; also the blood vessels are congested (red arrows). Hesperidin (100mg/kg) (**c**) showing normal structure of cerebral cortex formed of mostly normal neuron cells typically have large pale nuclei with prominent nucleoli; others still have pyknotic nuclei (green arrows); also astrocytes have normal looking; paler more elongated nuclei with scant cytoplasm and normal looking blood vessels. Hesperidin (200mg/kg) (**d**) showing normal structure of cerebral cortex formed of neuron cells typically have large pale nuclei with prominent nucleoli. The non-neuronal cells (glial) include oligodendrocytes; hyperchromatic round nuclei with abundant clear cytoplasm, astrocytes; paler more elongated nuclei with scant cytoplasm (H&E ×200)

A photomicrography of brain tissue in normal control group revealed normal histopathological picture. Brain tissue sections from OVA group showed paler astrocytes and pyknotic nuclei in neuron cells with congested blood vessels. Brain tissue section of a rat pretreated with hesperidin (100 mg/kg) showed normal structure of cerebral cortex, normal looking astrocytes, and normal looking blood vessels, while rats that were pretreated with hesperidin (200 mg/kg) revealed a marked improvement in the looking architecture of brain tissues (Fig. 11).

## Discussion

Asthma is a recurrent obstructive airway inflammatory condition attributable to inflammation and hypertrophy of airway leading to reduced lung function (Périz et al., 2020). Asthma has been reported to be difficult to treat due to the intricacy of its etiology, and that understanding the underlying mechanisms is critical in order to develop highly effective drugs with minimal side effects (Ma et al., 2019). There are many parallels between the OVA-triggered airway inflammation model and the symptoms of human allergic asthma (Maslan and Mims, 2014). Epidemiological studies revealed that those with asthma were 78% more likely to have mild cognitive impairment (Kroll et al., 2018). Thus, the present study was planned to assess the protective role of hesperidin against OVA-induced bronchial asthma and depression in rats.

Recently, the bioflavonoid hesperidin, also known as hesperetin 7-O-rutinoside or hesperitin-7-rhamnoglucoside, is primarily produced by many citrus trees as a secondary metabolite and is widely distributed in fruit juices (Lv et al., 2015). Bioflavonoids have attracted the attention of most researchers due to their unlimited biological importance (Iglesias et al., 2007). Citrus products like lemons (*Citrus limon*), sweet oranges (*Citrus sinensis*), bitter oranges (*Citrus aurantium*), citrons (*Citrus medica*), clementines (*Citrus clementina*), and mandarins (*Citrus reticulata*) are abundant sources of hesperidin (Meneguzzo et al., 2020)

Intriguingly, the current research showed that asthmatic control rats exposed to the OVA challenge exhibited depressive behavior, as evidenced by decreased activity count, locomotor activity, and increased immobility in FST. Patients with asthma often experience depression symptoms, which may elevate their chance of developing asthma-related morbidity and mortality (Loerbroks et al., 2012). Our findings are matching with the previous study conducted in mice-received OVA + Al (OH)3 that showed a marked decrease in the count of rearing (Conrad et al., 2009). Patients with asthma are more likely to experience behavioral cognitive alterations that could deleteriously affects their life (Hedges et al., 2019). Hesperidin successfully reduced the depressed behavior caused by OVA, as it could increase the activity count, locomotor activity, and decreased immobility in forced swimming test. This notion was supported by previous study showing that administration of hesperidin to rats suffering from Huntington’s disease could greatly improve the behavioral indices, due to its ability to restore the functions of the mitochondrial enzyme complex and reduce free radicals formation (Li et al., 2015). Furthermore, hesperidin could effectively improve the behavior of rats in depression model by utilizing chronic unpredictable mild stress model and showed anti-depression effect, due to its role in regulating the hypothalamic-pituitary-adrenal axis (Cai et al., 2013).

The definitive mechanism of the airway inflammatory response in asthma is dependent on activating the inflammatory cells, including lymphocytes, basophils, macrophage, and eosinophils, followed by synthesis and release of many pro-inflammatory mediators (Lukacs, 2001). Eosinophils are considered the chief modulator in the pathophysiology of bronchial asthma, where they trigger the recruitment of T cells to the lungs (Possa et al., 2013). Th2 lymphocytes are the main orchestrators of in airway inflammatory response through controlling the release of il-5 and TNF-α (Moldoveanu et al., 2008). TNF-*α* is a powerful pro-inflammatory cytokine that possess a vital role in modulating the immune response and airway inflammation (Zelová and Hošek, 2013). Moreover, TNF-α association with depression also seems to be verified (Dowlati et al., 2010). The findings showed a marked increase in TNF-*α* levels in the lungs of rats that received OVA, where high levels of TNF-*α* perform primarily on the smooth muscle cells within the airways, leading to leakage and damage to bronchial epithelial cells, and could exacerbate inflammatory and pro-oxidative function (Van Lieshout et al., 2009). This result is in agreement with previous study that showed that increased expression of TNF-*α* in the bronchoalveolar lavage fluid of asthmatics (Catal et al., 2015). Conversely, pre-treated rats with hesperidin, especially 400 mg/kg, could reduce the levels of TNF-α in the lungs of rats that received OVA. Previous results confirmed the anti-inflammatory role of hesperidin (Tejada et al., 2018, Ganeshpurkar and Saluja, 2019). Tanaka and Takahashi (2013) published that people especially children with higher food containing flavonoids intake were less susceptible to asthma (Tanaka and Takahashi, 2013).

Our study was directed to evaluate the roles of IL-5 and IgE in the pathophysiology of bronchial asthma. IL-5 is a potent pro-inflammatory cytokine that regulates eosinophil maturation, proliferation, activation, and migration (Greenfeder et al., 2001). IL-5, in particular, is responsible for allergen-induced airway eosinophilia and bronchial hyperresponsiveness in sensitized guinea pigs (Mauser et al., 1993). Additionally, IgE is crucial in the beginning and development of the inflammatory cascade and consequently allergic responses (Sandeep et al., 2010). In the current study, a significant elevation in IgE and IL-5 levels in the lungs of rats that received OVA was observed, while pre-treated with hesperidin ameliorated this elevation suggesting its immuomodulatory effect (Forastiere et al., 2000, Enejoh et al., 2015). Furthermore, hesperidin as flavonoid aglycone has a distinctive role in the treatment of asthma via modulation of certain biomarkers such as reactive oxygen species and IL-5 production (Saini et al., 2022).

TGF-β1 is one of airway remodeling mediators which has a vital role in asthma and associated with vascular endothelial cell proliferation and angiogenesis (Asfour et al., 2020). In the current work, OVA induced airway structural changes evidenced by an elevation of TGF-β1 levels while hesperidin could decrease TGF-β1 levels and protected from these changes modulating airway remodeling. Moreover, it has been documented that il-10 possess a significant anti-inflammatory impact in suppressing the levels of TNF-α (Ogawa et al., 2008). The present study documented that OVA-induced decreased il-10. This comes in accordance with previous studies (Jang et al., 2012, Dong et al., 2014). Conversely, hesperidin effectively increased the values of il-10. Our results are in consistent with a study that showed the anti-inflammatory role of hesperidin via increasing the levels of the anti-inflammatory cytokine (il-10) (Choi et al., 2021). Furthermore, regulatory T cells (Treg; CD4+CD25+Foxp3+) are considered the main regulators of Th2-induced allergic responses that have been stated to inhibit the potentially harmful inflammatory and immune responses (Ray et al., 2010). Treg cells that express the fork head/winged helix transcription factor (Foxp3) are recognized to possess anti-inflammatory properties via the contact with the cells directly or assist in releasing anti-inflammatory cytokines (Miller et al., 2015). It has been stated that pediatric patients with bronchial asthma had considerably low values of CD4+CD25+Foxp3+ Treg (Lee et al., 2007). Our results exhibited that OVA instillation decreased the expression of CD4+CD25+Foxp3+ Treg. Previous study documented the decrease in expression of CD4+CD25+Foxp3+Treg in BALB/c mice sensitized by OVA and cholera toxin (Cheng et al., 2019). However, pre-treated rats with hesperidin could significantly increase the population of CD4+CD25+Foxp3+Treg in OVA-induced bronchial asthma and depression. Prior study documented the anti-allergic and anti-inflammatory effect of hesperidin in NC/Nga mice via upregulating the Treg population (Nagashio et al., 2013).

The pathogenesis of allergic bronchial asthma is defined by a complicated interaction between inflammatory cells, epithelia, smooth muscle cells, and neurons (Renz, 2001). Our study was the first to evaluate the release of NT-3, dopamine, and serotonin associated with depression. Previous study documented that NT-3 levels in peripheral blood cells of depressed patients with bipolar disorder were decreased in a symptom-dependent manner (Otsuki et al., 2008). Administration of hesperidin could modulate the release of NT-3, dopamine, and serotonin during OVA-induced bronchial asthma and depression in rats. Our findings are matching with previous studies that documented the anti-depressant effects of hesperidin (Feng et al., 2016, Fu et al., 2019). Moreover, regarding histopathological investigation, OVA exposure resulted in a significant inflammatory infiltration, pulmonary architecture distortion, and paler astrocyte. Such OVA-induced alterations have previously been documented (Wang et al., 2017). The current study’s findings demonstrated that hesperidin could decrease the pathological alterations in the tissues of the brain and lungs. These results provide additional evidence in support of the findings of biochemical studies.

## Conclusion

Our findings clearly showed that hesperidin have a protective role against OVA-induced asthma and depression in rats (Fig. 1). This favorable role may be at least partially mediated by its immunoregulatory and anti-inflammatory actions, via reducing the lung levels of TNF-α, IgE, and Il-5 as well as increasing the release of Il-10. In addition, hesperidin significantly reduced TGF-β modulating airway remodeling as well as augmented the expression of CD4+CD25+Foxp3+Treg expression. Thus, our results imply that hesperidin may be used as a powerful immunoregulatory agent for the prevention, and treatment of allergic asthma by enhancing Treg.

## Data Availability

Available upon request.

## Abbreviations

OVA: ovalbumin
Al (OH) 3: aluminum hydroxide
IgE: immunoglobulin
IL-5: interleukin-5
IL-10: interleukin-10
TGF-β: transforming growth factor
ZnCl_2_: zinc-chloride
MREC: Medical Research Ethics Committee
NRC: National Research Centre
I.p.: intraperitoneal
TNF-α: tumor necrosis factor-alpha
Foxp3: fork head/winged helix transcription factor
NT-3: neurotrophin-3
Treg: regulatory T cells
ANOVA: one-way analysis of variance
